# Exploring the factors associated with coronaphobia among physicians during the COVID-19 outbreak in Egypt

**DOI:** 10.1186/s41983-021-00357-6

**Published:** 2021-07-29

**Authors:** Mohamed Abdelghani, Mervat S. Hassan, Hayam M. Elgohary, Eman Fouad

**Affiliations:** 1 Psychiatry Department, Faculty of Medicine, Zagazig University, Zagazig, Egypt; 2 2015-2016 Hubert H. Humphrey Fellowship, Department of Psychology, Virginia Commonwealth University, Richmond, USA

**Keywords:** Coronaphobia, COVID-19 outbreak, Physicians, Egypt

## Abstract

**Background:**

Coronaphobia refers to intensified and persistent fears of contracting COVID-19 virus infection. This study aimed to evaluate the newly termed phenomenon, coronaphobia, and address its associated correlates among Egyptian physicians during the outbreak. A cross-sectional study, including a total of 426 Egyptian physicians working during COVID-19 outbreak, was conducted between March 1st and May 1st, 2020. The Fear of COVID-19 Scale (FCV-19S) and Hospital Anxiety and Depression Scale (HADS) were utilized for assessment of coronaphobia, and comorbid anxiety and depressive symptoms among physicians, respectively during the outbreak.

**Results:**

Moderate-to-severe symptoms of anxiety and depression were reported by 28% and 30% of physicians, respectively. Physicians experiencing higher levels of coronaphobia were more likely to be females, nonsmokers, having death wishes and/or self-harming thoughts, receiving insufficient training, dissatisfied with their personal protective equipment (PPE), and had colleagues infected with COVID-19 virus infection. Coronaphobia was positively correlated with anxiety (*r* = 0.59, *P* < 0.001) and depressive symptoms (*r* = 0.47, *P* < 0.001).

**Conclusions:**

Egyptian physicians experienced higher levels of coronaphobia, anxiety, and depressive symptoms during the COVID-19 outbreak. Yet, frontline physicians did not differ from their second-line counterparts regarding the aforementioned symptoms. Routine mental and physical assessment measures of medical staff should be implemented.

## Background

In December 2019, the coronavirus disease 2019 (COVID-19) outbreak caused by severe acute respiratory syndrome coronavirus 2 (SARS-Cov-2) had arisen in Wuhan, China [1]. World Health Organization announced the COVID-19 outbreak, as a global pandemic in March 2020. Yet, there is a lack of definite information about the nature and course of this novel, rapidly spreading virus [2]. The COVID-19 pandemic itself, subsequent lockdown, social life disturbances, and financial problems are negatively affecting mental health and well-being [3]. Indeed, there is increasing attention to the psychological impact of the COVID-19 pandemic at the research [4], on general populations [5], as well as healthcare professionals [6].

The medical staff are the frontline health care workers who are facing excess load during the pandemic [2]. Excessive worries of COVID-19 were evident among the medical staff [7]. Coronaphobia, a novel term in the psychiatric literature, was reported in previous research worldwide, and likely related to the novelty of the virus and uncertainties about the consequences of the COVID-19 pandemic [8]. Coronaphobia refers to excessive fears and worries of being infected by coronavirus [9]. For the health care workers, direct contact with COVID-19 patients, limited numbers of protective equipment, and denying of COVID-19 virus infection by some patients are factors that increase these fears among medical staff about being infected with the virus and transmitting the infection to their families.

Moreover, the psychological responses to previous epidemics and pandemics suggest that different psychological vulnerability factors may play a role. The intolerance of uncertainty, perceived vulnerability to disease, anxiety proneness, lack of information, and getting misinformation from popular media headlines may, collectively or separately, play a substantial role in increasing health-related fears and phobias and shaping coronophobia [10]. This prolonged struggle may lead to anxiety and depression among the health care workers, especially physicians [11].

To our knowledge, studies examining and focusing on coronaphobia among medical staff during the COVID-19 outbreak in Egypt are scarce, if any. This study aimed to explore the newly termed phenomenon, coronaphobia, and identify its associated correlates among physicians during their battle against the COVID-19 pandemic in Egypt.

## Methods

The cross-sectional survey study was conducted from March 1st to May 1st, 2020. A total of 444 physicians, working during the COVID-19 outbreak in general hospitals in Sharkia Province, were included in the study. The sample size was calculated using Epi Info 6.0, at 80% power of the study, 95% confidence level [12]. In Sharkia Province, around 7760 physicians from various specialties are currently working in general hospitals [13]. As the local health authorities recommended social distancing, and owing to lockdown restrictions, the authors had designed a Google form including a questionnaire to collect sociodemographic and clinical data of participants, Fear of COVID-19 and Hospital Anxiety and Depression Scales for assessment of fear of COVID-19 levels and depression and anxiety respectively, and sent the corresponding link by Whats app. messages through telephone to the selected participants.

The physicians were chosen via the systematic random method. The largest three governmental general hospitals in Sharkia Province were identified where a random sample of physicians from each hospital was selected. By using physician official registry in the medical syndicate of Sharkia Province, name lists (files) of physicians of all specialties and all titles (residents, specialists, and consultants) in the records of each of the defined three hospitals were identified and numbered, then the authors randomly selected one physician based on a system of intervals (every fixed number), and the Google form was sent to each physician via Whats app. message.

During the data collection, eighteen physicians (4.1%) refused to complete the study. After excluding the incomplete responses, the final sample size included in the study was 426 physicians.

All participants were requested to complete the following measures:

A questionnaire including a semistructured checklist designed to collect relevant demographic and clinical data, included close-ended questions with dichotomous responses, either yes or no, investigating the lifetime history of mental illness namely depression, anxiety disorders, and self-harm thoughts and behaviors.

### Fear of COVID-19 Scale (FCV-19S)

This 7-item self-rating tool was utilized for assessing the severity of fears and apprehensions of contracting COVID-19 virus infection (coronaphobia). This scale entails 7 questions answered as “strongly disagree,” “disagree,” “neither agree nor disagree,” “agree,” and “strongly agree” with scores from 1 to 5 for each. The higher the score, the greater the level of coronaphobia. An Arabic version of this scale, examined for its reliability and validity, was used in this study [8, 14, 15].

### The Hospital Anxiety and Depression Scale (HADS)

This 14-item self-rating scale was used for screening the anxiety and depressive symptoms among the participants [16]. This scale included seven questions asking about depressive symptoms, and the other 7 asking for anxiety symptoms with a score of each question from 0 to 3. The overall score in each subscale ranged from 0 to 21. Total scores from 0 to 7 considered normal (non-cases), while scores from 8 to 10 were considered borderline (mild cases), and those above 11 were considered valid cases. An Arabic validated version of this scale was used in this study [17].

The data, in this study, were collected and analyzed using the Statistical Package for the Social Sciences (SPSS, Inc) software version 20.0 (Chicago IL, USA) [18]. Qualitative variables were described by frequency distributions, while quantitative variables were described by the means and standard deviations. The chi-squared test was used to compare the proportions of categorical data, and the independent sample *t* test was used to compare the means of two groups. To assess the relationship between two variables with a linear relationship, the Pearson correlation coefficient was used. All results were considered statistically significant when their significant probability was less than 5% (*P* < 0.05).

## Results

This study included a total of 426 Egyptian physicians who were working in general hospitals in Sharkia Province, 282 (66.2%) females, 152 (35.7%) residents, 139 (32.6%) working in private clinics or hospitals besides their governmental work, and 38 (8.9%) smokers. The means of age, daily working hours, and weekly working days were 34.57 ± 6.5 years, 8.15 ± 3.89 h, and 4.52 ± 1.48 days, respectively. Frontline physicians, who treated isolated patients diagnosed with COVID-19 virus infection, represented 15.5% of the physicians (*n* = 66), as shown in Table 1.

**Table 1:** Association between the score of FCV-19S and physicians' demographic and clinical characteristics

Demographic variables (*N* = 426)	FCV-19S^a^					
	*N*	%	Mean	SD	*t* test	*P*
Gender						
Male	144	33.8	18.52	4.97	− 4.692	< 0.001
Female	282	66.2	20.89	4.91		
Marital status						
Married	303	71.1	20.04	5.09	0.318	0.751
Not married	123	28.9	20.21	4.96		
Professional title						
Resident	152	35.7	20.53	4.93	1.332	0.185
Specialist/consultant	274	64.3	19.85	5.11		
Daily working hours						
≤ 8	258	60.1	20.04	5.18	− 0.435	0.664
> 8	119	39.9	20.28	4.77		
Working days/week						
≤ 5	274	64.5	20.45	5.05	1.915	0.056
> 5	152	35.5	19.47	5.02		
Received sufficient training						
No	310	72.8	20.54	5.08	3.032	0.003
Yes	116	27.2	18.89	4.79		
Satisfied with hospital PPE**						
No	358	84.0	20.51	5.00	3.972	< 0.001
Yes	68	16.0	17.90	4.78		
Physician's role						
Frontline	66	15.5	20.18	5.35	− 0.162	0.871
Second-line	360	84.5	20.07	5.00		
History of colleague affected with Covid-19						
No	239	56.1	19.64	5.19	− 2.063	0.040
Yes	187	43.9	20.66	4.82		
History of chronic mental illness						
No	316	74.2	19.85	4.86	− 1.629	0.104
Yes	110	25.8	20.76	5.54		
History of chronic medical illness						
No	335	78.6	19.90	4.92	− 1.497	0.135
Yes	91	21.4	20.79	5.48		
Smoking status						
No	388	91.1	20.26	5.04	2.177	0.030
Yes	38	8.9	18.39	4.87		
History of substance use						
No	412	96.7	20.05	4.94	− 0.955	0.340
Yes	14	3.3	21.36	7.82		
History of self-harm thoughts during pandemic						
No	383	89.9	19.88	4.98	− 2.570	0.011
Yes	43	10.1	21.95	5.33		

^a^ Fear of COVID-19 Scale. ^b^ Personal protective equipment

**Fig. 1:**
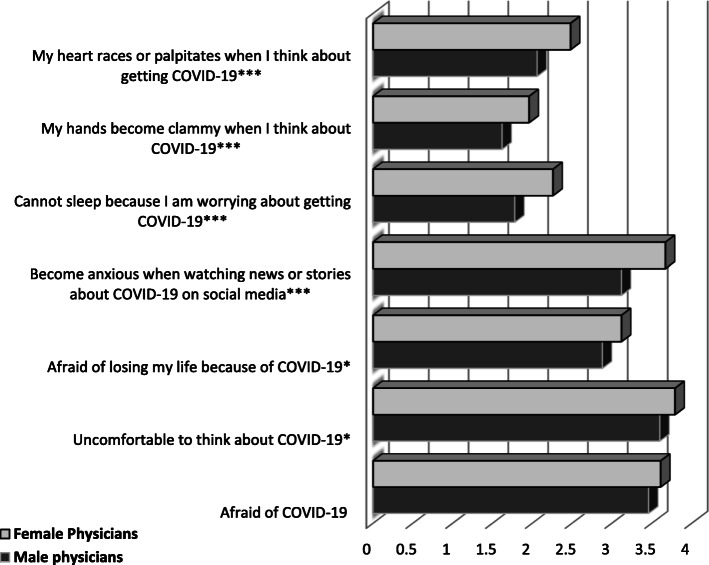
Distribution of items of FCV-19S by physician's gender. * *P* < 0.05, ** *P* < 0.01, *** *P* < 0.001. *COVID-19* coronavirus disease 2019, *FCV-19S* Fear of COVID-19 Scale

Of note, 73% (*n* = 310) and 84% (*n* = 358) of physicians reported receiving insufficient training related to pandemic and dissatisfaction with the hospital’s personal protective equipment (PPE), respectively. Regarding the psychiatric symptoms, death wishes and/or self-harming thoughts, moderate-to-severe anxiety, and depressive symptoms were reported by 10%, 28%, and 30% of physicians, respectively. Female physicians, compared with their male counterparts, reported higher levels of fear of COVID-19 virus infection (coronaphobia) in six out of the seven items, as well as the overall score of the FCV-19 Scale, as shown in Fig. 1.

Table 1 illustrated the relationship between the demographic and clinical variables, and the mean score of coronaphobia (measured by the Fear of COVID-19 Scale). Physicians with higher levels of coronaphobia were more likely to be females (20.89 ± 4.91 vs 18.52 ± 4.97, *P* < 0.001). According to the recently adopted cut-off point for FCV-19S, it was found that the prevalence of coronaphobia among the selected physicians was 75.4% (*n* = 321), nonsmoker (20.26 ± 5.04 vs 18.39 ± 4.87, *P* = 0.03), having death wishes and/or self-harming thoughts (21.95 ± 5.33 vs 19.88 ± 4.98, *P* = 0.011), receiving insufficient training related to the pandemic (20.54 ± 5.08 vs 18.89 ± 4.79, *P* = 0.003), dissatisfied with the hospital PPE (20.51 ± 5.0 vs 17.90 ± 4.78, *P* < 0.001), and having a history of colleagues who were affected with COVID-19 infection (20.66 ± 4.82 vs 19.96 ± 5.19, *P* = 0.04), nonsmoker (20.26 ± 5.04 vs 18.39 ± 4.87, *P* = 0.03). Similarly, coronaphobia was found to be positively correlated with the mean scores of both symptoms of anxiety (*r* = 0.59, *P* < 0.001) and depressive symptoms (*r* = 0.47, *P* < 0.001), as illustrated in Table 2.

**Table 2:** Correlation between the score of FCV-19S and severity of anxiety and depressive symptoms among physicians

*N* = 426		FCV-19Sa	
	M (SD)	Pearson correlation (*r*)	*P*
Age	34.57 (6.50)	− 0.084	0.085
HADS^b^			
Total Anxiety Score	8.05 (4.01)	0.588	< 0.001
Total Depression Score	9.07 (3.27)	0.465	< 0.001

^a^ Fear of COVID- 19 scale. ^b^ Hospital Anxiety and Depression Scale

Frontline physicians, compared with second-line physicians, were more likely to be non-married (39.4% vs. 26.9%, *P* = 0.04), residents (46% vs 33.6%, *P* = 0.037), working more than 8 h/daily (43.9% vs 24.7%, *P* = 0.001), received sufficient training related to the pandemic (37.9% vs 25.3%, *P* = 0.035), and have a history of medical colleague affection with COVID-19 infection (69.7% vs 39.2%, *P* < 0.001), as illustrated in Table 3.

**Table 3:** Comparison between frontline and second-line physicians regarding their demographic and clinical characteristics

*N =* 426	Frontline (*n* = 66)	Second-line (*n* = 360)	*t X*^*2*^	*P*
	*N* (%), M (SD)			
Age	33.24 (6.57)	34.81 (6.49)	1.802	0.072
Gender				
Male	26 (36.4)	118 (32.8)	1.091	0.296
Female	40 (60.6)	242 (67.2)		
Marital status				
Married	40 (60.6)	263 (73.1)	4.209	0.040
Not married	26 (39.4)	97 (26.9)		
Professional title				
Resident	31 (47.0)	121 (33.6)	4.337	0.037
Specialist/consultant	35 (53.0)	239 (66.4)		
Daily working hours				
≤ 8	37 (56.1)	268 (75.3)	10.265	0.001
> 8	29 (43.9)	88 (24.7)		
Working days/week				
≤ 5	45 (68.2)	228 (63.9)	0.453	0.299
> 5	21 (31.8)	129 (36.1)		
Received sufficient training				
No	41 (62.1)	269 (74.7)	4.469	0.035
Yes	25 (37.9)	91 (25.3)		
Satisfied with hospital PPE^b^				
No	57 (86.4)	301 (83.6)	0.315	0.362
Yes	9 (13.6)	59 (16.4)		
History of colleague affected with Covid-19				
No	20 (30.3)	219 (60.8)	21.110	< 0.001
Yes	46 (69.7)	141 (39.2)		
History of self-harm thoughts during pandemic				
No	57 (86.4)	326 (90.6)	1.080	0.299
Yes	9 (13.6)	34 (9.4)		
FCV-19S^a^	20.18 (5.35)	20.07 (5.00)	− 0.162	0.871
HADS^c^				
Total Anxiety Score	8.67 (4.31)	7.93 (3.67)	− 1.260	0.208
Total Depression Score	9.11 (2.84)	9.07 (3.35)	− 0.090	0.928

^a^ Fear of COVID- 19 scale. ^b^ Personal protective equipment. ^c^ Hospital Anxiety and Depression Scale

## Discussion

COVID-19 virus infection does not only affect physical health but also has an impact on the mental health especially of health care providers, who are on the front lines, as they are fighting their global long-drawn battle against COVID-19 [19]. Coronaphobia is a newly emerging phenomenon specifically generated to define excessive fears and worries of contracting COVID-19 virus infection. It has recently been replicated in the research of the mental health burden of the COVID-19 pandemic [10]. The main finding of this study is that the physician, in Egypt, experienced higher levels of coronaphobia during the COVID-19 pandemic. In line with this finding, numerous studies documented intensive and persistent fears and worries not only among physicians but also other health care providers including nurses, technicians, and health workers during the COVID-19 pandemic [2, 7]. The excess fear might be related to the initially insufficient understanding of the virus, the lack of prevention and control knowledge, the shortage and improper use of medical protective equipment, and the exposure to critical life events as the death of patients [20]. Also, as claimed by some of the health care providers, there was the fear of the possibility of the virus transmission to their families or close friends [21]. These concerns would lead the working physicians to isolate themselves from their family members, modify or even change their lifestyle routines, and limit their social connectedness which resulted in psychological pressure, feelings of loneliness and helplessness, and despair [22].

Work overload, burnout, and stress symptoms during the COVID-19 pandemic would make physicians more prone to developing psychiatric disorders like anxiety, depression, and suicide, which negatively affect their quality of life [23–25]. Consistent with this, our study found that 10%, 28%, and 30% of physicians reported thoughts of death or self-harming thoughts, intense anxiety, and depressive symptoms, respectively. Numerous studies had documented the intensified levels of anxiety and depression among health care professionals during the pandemic. A study stated that the prevalence rates of anxiety and depression among physicians were 25.67% and 28.13%, respectively [26]. During the COVID-19 pandemic, it was reported that more than 70% of the medical staff stated intense apprehension, 25% showed anxiety, and 12% presented with depression [2]. A study conducted at the early stages of COVID-19 in China found that 50% and 45% of health care providers had depression and anxiety symptoms, respectively [27], while another study stated that 14% and 24% of the medical staff experienced depression and anxiety with COVID-19, respectively [28]. The discrepancies in the prevalence of depression and anxiety among the studies may be related to variance in sample size, different assessment tools used, different cultural backgrounds, and different stages related to the pandemic onset.

As illustrated in Table 2, there was no significant correlation between age and coronaphobia. In line with this finding, Ahorsu et al. (2020), the founders of the FCV-19S, stated that age appeared not to affect the response pattern of the fear on the FCV-19S [14]. This study found physicians with higher levels of coronaphobia who were more likely to be females, receiving insufficient training related to the pandemic, dissatisfied with the hospital PPE, and having medical colleagues who were infected with the COVID-19 virus infection. These findings were in line with other studies which also found that females have been reported higher levels of anxiety than males [21, 26], yet, due to the self-reporting nature of most scales used for assessing anxiety, some argued male participants might have minimized symptoms [22]. Studies of previous pandemics and outbreaks also revealed that inadequate staff training [29], inadequate organizational support [30], frustration about the efficacy of precautionary measures [31], being less experienced [32], and lack of confidence in infection control and caring for affected medical colleagues during the pandemic [33] were the main variables increasing the risk of psychological distress including anxiety, depression, and stress among health care workers. Furthermore, it was claimed that severe anxiety symptoms experienced among physicians during their duty were related to their suspicion of being infected with the COVID-19 virus [1].

Integration between physical health (body) and mental health (mind) is essential to the current psychological state of physicians. In this study, the relationship between coronaphobia and the associated psychological symptoms cannot be ignored. Physicians with high levels of coronaphobia were more likely to have death wishes and/or self-harming thoughts as well as more anxiety and depressive symptoms. In line with our findings, excessive anxiety and fears of COVID-19 virus infection were found to be associated with suicidal thoughts and intense feelings of hopelessness [34]. Moreover, it was reported that during the pandemic, the prolonged worries would increase levels of anxiety, depression, and stress in individuals or increase the severity of the pre-existing psychiatric symptoms [35]. Similarly, the higher the scores on the fear of the COVID-19 virus scale, the higher the levels of anxiety and depressive symptoms measured by the Hospital Anxiety and Depression Scale (HADS) [14]. These replicated results might suggest that physicians with severe fear of COVID-19 might have been suffering or being at greater risk to develop these comorbid disorders. It was found that comorbid physical health conditions, history of mental health disorders, and substance use were significant risk factors of stress, depression, and anxiety among health care providers during the COVID-19 pandemic [1]. However, this study failed to find a relationship between increased scores of coronaphobia and histories of mental, medical, or substance use disorders among physicians. These findings could be rationalized as being a conservative community where mental disorders and substance use were associated with stigma, which might lead some participants to deny having mental or substance use disorders. Also, most of the participants in our study were young adults (mean age = 34.57 years), we can argue that chronic medical diseases are not common at this age (only 21% had a medical disease). Of interest, physicians who were smokers reported lower levels of coronaphobia in this study. It was claimed that nicotine smoking could be viewed as a remedy that acutely reduced anxiety symptoms, especially during stressful situations [36]. It also could be related that they usually have less trait anxiety, as a consequence of blunting the sympathoadrenal axis [37].

Frontline physicians are the ones currently working with COVID-19 patients. Forty-seven percent of the frontline physicians were residents, while 66.4% of the second-line physicians were specialists and consultants. In Egypt, residents of all specialties and medical officers were usually at the frontline (for example in emergency rooms). They were in direct contact and manage the patients before referral to their 2nd on-call supervisors (specialists and consultants). This would explain why most consultants were located in the second-line hospital departments.

The frontline physicians caring for COVID-19 patients, compared with their second-line counterparts, were supposed to experience higher levels of anxiety and depressive symptoms, and sleep disturbances [27]. As expected, owing to the nature of their current work duties, this study found that frontline physicians were more likely to be residents, working more than 8 h daily, received sufficient training related to the pandemic and have a history of medical colleague affection with COVID-19 infection when compared with their second-line counterparts. However, although the mean scores of coronaphobia, anxiety, and depressive symptoms among frontline physicians were higher than those of their second-line counterparts, the difference was not statistically significant. Consistent with our results, in terms of excessive fears and depressive symptoms, a recent study did not find any differences between the medical staff at the frontline areas (working in the respiratory, emergency, ICU, and infectious diseases departments) and those with lower risks of contact with COVID-19 patients (second-line medical staff working in other departments) [7]. Similarly, another study found no differences in self-rated anxiety and depressive symptoms among the staff in the COVID-19–associated department and other departments [38]. This lack of significant differences between the frontline and second-line staff was probably attributed to several reasons. First, second-line physicians might experience feelings of sympathy (towards both COVID-19 patients and frontline physicians), guilt, isolation, and apparent feeling of worthlessness due to the inability to share in the present crisis [39]. Second, with increasing admissions of COVID-19 patients and shortage of medical staff, the second-line physicians might be worried about being allocated by the government to work in the frontline areas despite being insufficiently trained or qualified to work with COVID-19 patients [40]. Third, their fears might be fueled by the floods of information from TV and media about suffering, illness, and the death of their frontline colleagues. All these feelings might in turn lead to the intensification of symptoms of fear, anxiety, and depressive symptoms among second-line physicians, and explain the absence of significant differences in the emergence of psychological symptoms when compared with their frontline counterparts.

The results of the current study should be interpreted in the context of its limitations. First, because of the disease outbreak, we were unable to conduct face-to-face interviews, instead, depended on self-rating scales with response bias, so these scales only provided a preliminary screening; thus, further confirmation and intervention were warranted. Second, the selection of participants from Sharkia Province, despite being one of the largest provinces in Egypt, would limit the generalization of our findings. Third, being a cross-sectional study might limit the cause–effect relationship. So, further future longitudinal studies are needed. In addition, it was difficult during the pandemic to include a control group of nonmedical personnel. Although these limitations were present, to our knowledge, this study would be the first in Egypt and the Middle East to assess the psychological impact of the COVID-19 virus infection among physicians using a new specific assessment tool for evaluating the corona virus-related fears.

## Conclusions

During the COVID-19 pandemic, physicians had higher rates of coronaphobia which in turn might lead to or intensify anxiety and depressive symptoms among them; however, frontline physicians were not significantly different from second-line physicians regarding rates of these symptoms. Female gender, receiving insufficient training related to the pandemic, dissatisfaction with the hospital PPE measures, and having a colleague affected with COVID-19 infection were significant risk factors for coronaphobia among physicians. Protecting health care providers is a central component of public health policy during the COVID-19 pandemic. Routine mental and physical assessment of medical personnel involved in diagnosing and treating patients with COVID-19 should be guaranteed, and special interventions to promote their mental well-being, when affected, need to be implemented.

## Data Availability

All datasets, generated or analyzed in this study, are available from the corresponding author upon reasonable request.

## Abbreviations

COVID-19: Coronavirus disease
FCV-19S: Fear of COVID-19 scale
HADS: Hospital anxiety and depression scale
PPE: Personal protective equipment
SARS-Cov-2: Severe acute respiratory syndrome coronavirus 2
